# Influence of Strict Lockdown on Vitamin D Deficiency in Pregnant Women: A Word of Caution

**DOI:** 10.3390/nu15081972

**Published:** 2023-04-19

**Authors:** Nuria Agüero-Domenech, Eduardo Bernabeu, Antonio García-Valentín, Ana Sarrión, Silvia Jover, Javier Baranda, Ernesto Cortés-Castell, Vicente Gil-Guillén, María J. García-Teruel

**Affiliations:** 1 Department of Gynaecology and Obstetrics, Hospital General Universitario Elda, 03600 Elda, Spain; nuria.aguero@umh.es (N.A.-D.); sarrion_ana@gva.es (A.S.); jover_sil@gva.es (S.J.); baranda_jav@gva.es (J.B.); garcia_mjter@gva.es (M.J.G.-T.); 2 Department of Public Health, History of Science and Gynaecology, Miguel Hernández University, 03550 San Juan, Spain; 3 Department of Cardiac Surgery, Hospital General Universitario Dr. Balmis, 03010 Alicante, Spain; antonio@garciavalentin.es; 4 ISABIAL (“Instituto de Investigación Sanitaria y Biomédica de Alicante”), 03010 Alicante, Spain; 5 Department of Pharmacology, Pediatrics and Organic Chemistry, Miguel Hernández University, 03550 San Juan, Spain; ernesto.cortes@umh.es; 6 Department of Clinical Medicine, Miguel Hernández University, 03550 San Juan, Spain; vgil@umh.es

**Keywords:** quarantine, 25(OH)D concentration, vitamin D deficiency, irradiation, pregnant women

## Abstract

The main source of vitamin D results from skin sunlight exposure. Vitamin D deficiency (VDD) is linked to several adverse events during pregnancy. While performing a cross-sectional study with 886 pregnant women in Elda (Spain) from September 2019 to July 2020 to determine the association of VDD with gestational diabetes mellitus in relation to body mass index, a strict lockdown (SL) due to the COVID-19 pandemic was declared from 15 March 2020 to 15 May 2020. To determine if VDD prevalence in the local population of pregnant women was influenced by SL, a retrospective cross-sectional study was conducted to estimate the prevalence odds ratio (POR) for the association of VDD and SL. A crude logistic regression model was calculated, and then further adjusted by the biweekly measured vitamin D-specific UVB dose in our geographical area. The POR during SL was 4.0 (95%CI = 2.7–5.7), with a VDD prevalence of 77.8% in the quarantine period. Our results revealed that VDD prevalence in pregnant women was influenced by SL. This valuable information could guide us in future if public officials order the population to stay indoors for any given reason.

## 1. Introduction

The main source of vitamin D is a result of the skin receiving a UVB portion of sunlight exposure. The amount of UVB radiation depends on several variables, such as solar zenith angle, latitude, season, and time of day [1,2,3]. There have been several studies that have explored the relationship between UVB radiation exposure and serum 25-hydroxyvitamin D (25(OH)D) concentrations in humans [4,5,6]. Recently, it has been described that there are significant seasonal and regional differences in UVB radiation across Europe, which can have an impact on vitamin D production in the human body. Furthermore, monthly differences in cumulative and weighted UVB radiation, which take into account factors such as latitude, altitude, and cloud cover, are significantly correlated with changes in mean 25(OH)D levels in different populations [7].

In adults, vitamin D deficiency (VDD) is defined as a 25(OH)D serum level below 20 ng/mL, and vitamin D insufficiency as a level between 20 and 30 ng/mL [8]. One of the primary causes of VDD is inadequate exposure to sunlight [9]. VDD has been observed in all ages, genders, and regions, including in the sunny Mediterranean regions [10]. In Europe, the reported prevalence of VDD in pregnancies is as high as 57% [11], but there is no agreement on the necessity of assessing 25(OH)D and the requirement for supplementation during pregnancy [12].

VDD has been associated with several adverse outcomes in pregnancy, such as pre-eclampsia, gestational diabetes mellitus (GDM), preterm birth, and caesarean delivery [12,13,14]. Rostami M. et al. reported the efficacy of a prenatal screening programme to improve 25(OH)D levels in pregnancy, and found a remarkable reduction in adverse pregnancy outcomes in women who were screened and supplemented with vitamin D [15,16].

The COVID-19 pandemic required governments around the world to adopt special measures to control social interaction. Recently, the effects of COVID-19 home confinement have been observed in relation to eating behaviour and physical activity [17]. In Spain, a strict lockdown (SL) was declared, with the population being confined at home, therefore influencing their exposition to sunlight. While investigating the relationship between VDD and GDM in a population of pregnant women, a proportion of them suffered as a result of the SL. This circumstance gave us the opportunity to describe how this enforced confinement influenced VDD prevalence. To adjust for 25(OH)D levels by sunlight irradiation in our study participants, we gathered the data of the vitamin D-specific UVB (D-UVB) dose received in the geographical area during the same period as the study [7].

The main objective of this research was to establish whether the prevalence of VDD in pregnant women was influenced by SL in Spain.

## 2. Materials and Methods

### 2.1. Study Population

A single-centre, population-based, observational, cross-sectional, and analytical study was carried out from September 2019 to August 2020 to investigate the relationship between VDD and GDM in relation to body mass index (BMI). Inclusion criteria and sampling were previously described. The project was approved by the Institutional Review Board of the Hospital General Universitario de Elda, and all subjects gave informed consent prior to inclusion [18].

Due to the COVID-19 pandemic and imposed measures by the Spanish Government, a significant cohort of participants experienced a strict lockdown in their geographical area (from 15 March 2020 to 15 May 2020). Therefore, we performed an additional analysis in a retrospective fashion to describe the impact of quarantine on the prevalence of VDD in the pregnant women population. 

### 2.2. Data Collection

Data were collected over a period of 10 months using anonymised data forms, on a retrospective and prospective basis, depending on the variable. To complete the said retrospective study, two new variables were defined: Strict Lockdown (SL) group and vitamin D-specific UVB (D-UVB) dose.

#### 2.2.1. Evaluation of Vitamin D Levels

Blood was sampled during the second trimester routine visit, in conjunction with a screening test for GDM. The serum 25(OH)D concentration was measured by an electrochemiluminescent automated binding assay (Modular Analytics E170 and Elecsys Vitamin D Total II, Roche Diagnostics, GmBH (Manheim, Germany)), with a measuring range from 3 to 70 ng/mL. This assay has been previously validated by liquid chromatography–tandem mass spectrometry (LC/MS/MS), and has been accredited by the Vitamin D Standardisation and Certification Program (VDSCP) of the CDC [19,20,21,22,23]. VDD was established as a serum concentration <20 ng/mL [8,24].

#### 2.2.2. Covariate Assessment

At the first trimester follow-up visit, a medical history and physical assessment were completed, and socio-demographic and pregnancy-related data were collected, such as maternal age (in years), smoking, hypothyroidism, ethnicity, weight (in kg), height (in m), parity, and history of caesarean section.

In the second trimester, coinciding with the 20-week ultrasound, a survey (San Carlos Study questionnaire) was completed to assess physical activity, nutritional habits, and lifestyle, presented in the form of scores. This questionnaire is based on the evidence-based nutritional recommendations of the American Diabetes Association (ADA), adapted to the Spanish population following the Diabetes Nutrition and Complications Trial (DNCT), and has been validated in our population [25,26]. Routine second trimester blood tests included the following: C-reactive protein (CRP) (in mg/dL), ferritin (in mg/dL), total cholesterol (in mg/dL), HDL cholesterol (in mg/dL), LDL cholesterol (in mg/dL), triglycerides (in mg/dL), fibrinogen (in mg/dL), haemoglobin (in g/dL), and haematocrit (in %).

BMI was calculated from weight (in kg) and height (in m) registered at the first trimester visit of pregnancy. On the basis of BMI, women were grouped as normal (<25), overweight (25–30), and obese (>30), following the World Health Organization’s classification.

#### 2.2.3. Definition of Strict Lockdown (SL) Group

All participants whose blood samples for vitamin D levels were taken from 15 March 2020 to 15 May 2020 were categorised as the SL group. The rest of the participants were categorised as the non-Strict Lockdown group (NSL). 

According to the Spanish Government decree, a lockdown was imposed on 14 March 2020. It was announced that, as of the following day, all non-essential workers were required to stay at home for the next 14 days. This situation was extended consecutively until 15 May 2020. Citizens were not allowed to leave their homes, except in the case of essential workers, and for the purchase of foodstuffs and basic goods.

#### 2.2.4. Assessment of Vitamin D-Specific UVB Dose in Study Geographical Area

The Tropospheric Emission Monitoring Internet Service (TEMIS) is part of the European Space Agency (ESA) Data User Programme. The TEMIS project generates datasets of several key information items, and provides access to them free of charge via the internet (www.temis.nl/uvradiation/UVdose.html (accessed on 10 February 2023); version 2.0) [27]. D-UVB is the effective UV irradiance (expressed in kJ/m^2^) reaching the Earth’s surface integrated over the course of the day, and was calculated by considering the UVB radiation dose at the wavelengths that can induce cutaneous vitamin D production (290–315 nm) [7]. This database has been already used in the past and is described in detail elsewhere [28,29].

The daily D-UVB doses were obtained for all three geographical coordinates available in the TEMIS database, corresponding to our study area, from 16 September 2019 to 15 August 2020 ([1]: latitude 38.375 and longitude −0.875; [2]: latitude 38.375 and longitude −0.620; [3]: latitude 38.625 and longitude −0.875), and the daily average of all of them was used for calculations. A cloud-modified dataset was used to account for the effects of clouds in the D-UVB radiation measurements. For each fortnight in the study, the average D-UVB dose was calculated. All participants were assigned a dose based on the fortnightly period in which the 25(OH)D assessment was performed.

### 2.3. Statistical Analysis

A descriptive analysis examined the distribution of the studied variables, with frequency calculations for qualitative variables, and means and standard deviations for continuous variables. A pairwise methodology was used for the management of missing data. For socio-demographic variables, 95% confidence intervals were calculated using Wilson’s method for proportions and the asymptotic method for continuous variables. Contingency tables were used to evaluate the factors associated with SL and VDD. Qualitative variables were compared using the chi-square test, and for quantitative variables the Student’s *t*-test was used.

A cross-sectional study was conducted to estimate the prevalence odds ratio (POR) for the association of VDD and SL in our pregnant women population. To control the influence of seasonal variations in sunlight irradiation, the logistic regression model was adjusted by the biweekly measured D-UVB dose in our geographical area. Covariates were explored as confounding factors and multivariate analyses were performed to determine the simplest explicative model.

The analyses were all carried out with STATA 14 (StatCorp LLC, College Station, TX, USA) and IBM SPSS v.26 (IBM Corp., Armonk, NY, USA). The figures were plotted using Microsoft Excel for Mac (Microsoft Corp., Redmond, WA, USA).

## 3. Results

### 3.1. Characteristics of the Study Participants

A full sample of 886 participants were included in the study period, but serum 25(OH)D levels were only obtained from 881 of them. Table 1 describes the characteristics of the participants in relation to their strict lockdown status. Roughly, 1 in 5 pregnant women experienced the strict lockdown (19%).

In the univariate analysis (Table 1), the participants were compared according to SL status. The SL group showed lower mean values for 25(OH)D (14.2 ng/mL vs. 20.5 ng/mL, *p* < 0.01) and a higher prevalence of VDD (77.8% vs. 50.1%, *p* < 0.01) compared to the NSL group. In addition, in the comparison of continuous variables, pregnant women in the SL group presented higher levels of triglycerides (193.5 mg/dL vs. 173.4 mg/dL, *p* < 0.01), and lower scores in the nutritional (4.1 vs. 3.2, *p* < 0.05) and lifestyle (3.3 vs. 2.3, *p* < 0.01) aspects of the St. Carlos Study questionnaire. In the qualitative analysis, there were no significant differences in other variables, including prevalence of GDM or distribution by BMI groups.

### 3.2. Distribution of Measured VD-UV Dose in Our Geographical Area 

**Figure 1:**
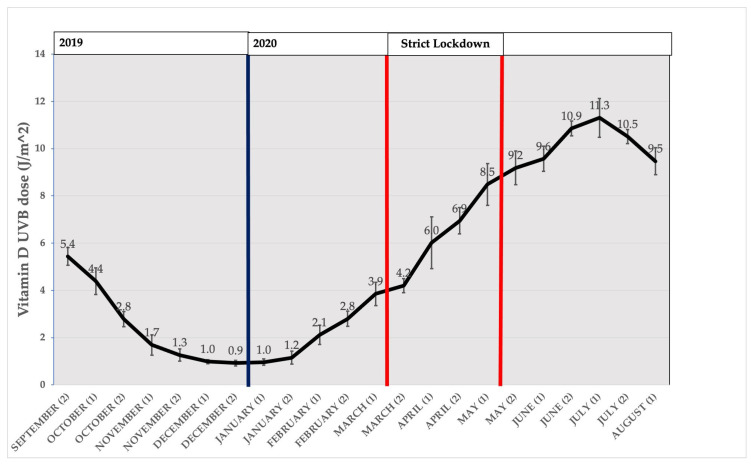
Distribution of vitamin D UVB dose (J/m^2^) by fortnight from September 2019 to August 2020, summarised as the mean of the days in each fortnight. Bars represent 95%CI. Dark blue bold vertical line represents the beginning of year 2020. Red bold vertical lines represent the beginning and the end of strict lockdown.

Figure 1 represents the distribution of mean biweekly D-UVB dose in our geographical area, as an average of three observation locations measured and reported in the cloud-modified TEMIS database, from mid-September 2019 to mid-August 2020. There was a decrease in the D-UVB dose by the end of the year, with the lowest point being reached in the second fortnight of December 2019, followed by a progressive increase in the D-UVB dose, reaching a peak by July 2020. “Vitamin D winter” has been defined as the threshold of D-UVB dose below which (<1 kJ/m^2^) vitamin D synthesis is negligible [7,30]. In the area of our study, this would correspond to the period from December to mid-January (lasting <2 months).

### 3.3. Seasonal Distribution of Average Measured Vitamin D

**Figure 2:**
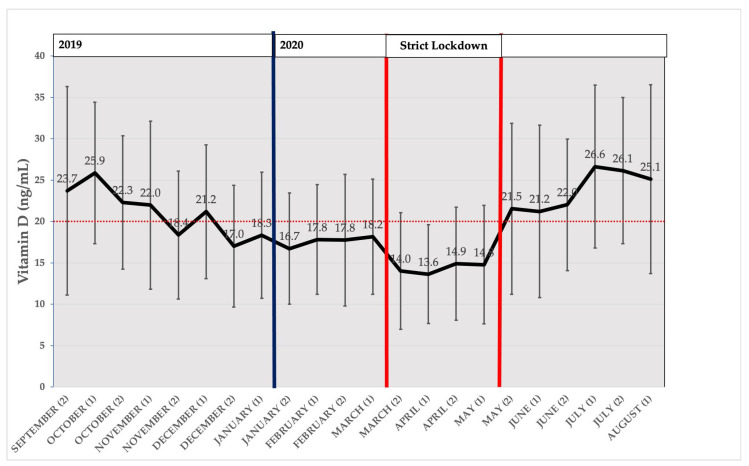
Distribution of vitamin D Levels (ng/mL) by fortnight from mid-September 2019 to mid-August 2020, expressed as mean 25(OH)D (in ng/mL) of all participants by biweekly intervals. Bars represent 95%CI. Dark blue vertical line represents the beginning of year 2020. Red bold vertical lines represent the beginning and the end of strict lockdown. The horizontal red dotted line represents the threshold for VDD diagnosis (20 ng/mL).

Figure 2 represents the distribution of the biweekly means of the 25(OH)D of all participants. While the distribution of means followed a seasonal pattern from September 2019 to mid-March 2020, there was a drop during the SL period (between red lines). After the quarantine ended, 25(OH)D levels returned to those expected. Mean 25(OH)D levels were lower in the strict lockdown period, even compared to those in “vitamin D winter”.

### 3.4. Seasonal Distribution of VDD Prevalence

**Figure 3:**
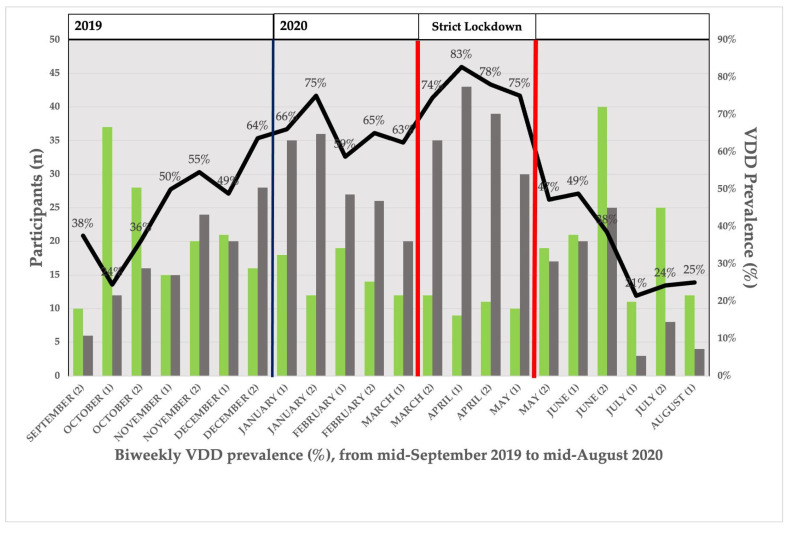
Distribution of VDD prevalence by fortnight from mid-September 2019 to mid-August 2020, expressed as percentage of participants with VDD by biweekly intervals (solid black line). Bars represent participants with 25(OH)D >20 ng/mL (green bar) or <20 ng/mL (grey bar) in each biweekly period. Dark blue vertical line represents the beginning of year 2020. Red bold vertical lines represent the beginning and the end of strict lockdown.

Figure 3 represents the distribution of VDD prevalence across the study period. The prevalence followed a seasonal pattern. Predictably, VDD prevalence increased with decreasing UVB irradiance. This prevalence was at its highest in relation to the “vitamin D winter”. With an increase in UVB irradiation, the prevalence gradually decreased. Nevertheless, the occurrence of a strict lockdown period between 15 March 2020 and 15 May 2020 was followed by a second increase in the prevalence of VDD. VDD peaked at 83% in the first half of April 2020. The prevalence of VDD in April 2020 exceeded that achieved in the expected “vitamin D winter” period, with a second “vitamin D winter”-like period appearing.

### 3.5. Regression Analysis of 25(OH)D Levels and D-UVB Dose in Relation to SL Status

To understand the relation of 25(OH)D levels with D-UVB dose, we performed a regression analysis and explored the impact of SL status. This analysis yielded the following coefficients: constant = 11.6 (*p* < 0.01), [D-UVB] dose = 0.4 (*p* < 0.01), and [SL] = −6.9 (*p* < 0.01). 

The regression equation was

25(OH)D (ng/mL) = 18.5 − 6.9 × [SL] + 0.4 × [D-UVB] (in J/m^2^) (1)

SL group: [SL] = 1, and NSL group: [SL] = 0

This result means that 25(OH)D levels were, on average, 6.9 ng/mL higher in the NSL group compared to the SL group (*p* < 0.01).

### 3.6. Cross-Sectional Study: Assessment of the Association of VDD and SL 

For an overall of 881 valid observations (99.4%), the prevalence of VDD was 55.5% (95%CI = 52.2–58.4%). In the SL cohort, the VDD prevalence was 77.8% (95%CI = 71.0–83.3%), while it was only 50.1% in the NSL cohort (95%CI = 46.5%–53.8%) (Table 1), with a prevalence odds ratio (POR) of 3.5 (95%CI = 2.4–5.1, *p* < 0.01) (Table 2).

### 3.7. Binary Logistic Model of VDD in Relation to SL Adjusted by Measured D-UVB Dose

The crude binary logistic model was further adjusted by D-UVB exposition to exclude seasonal bias (Table 2). All participants were assigned the D-UVB dose of the fortnight when the 25(OH)D levels were sampled. Once adjusted by biweekly means of the real measurements of D-UVB dose in our geographical area, the POR for SL rose to 4.0 (95%CI = 2.7–5.7) and the POR for the D-UVB dose was 0.9 (95%CI = 0.9–0.9). The rest of the covariates were explored as confounding factors in multivariant models, but no significant modification of the VDD POR or improvement in the precision of the confidence interval were obtained, with the bivariant model being most parsimonious.

The equation for the adjusted regression model, representing the probability of VDD, was as follows:

(2) $$P(\mathrm{VDD})=\frac{1}{1+e^{-\left(1.53+4.01\times \left[\mathrm{SL}\right]+0.91\times \left[D-\mathrm{UVB}\right]\;\left(\mathrm{inJ}/m^{2}\right)\right)}}$$

SL group: [SL] = 1, and NSL group: [SL] = 0

This finding means that the VDD prevalence in participating pregnant women was significantly influenced by SL. This observation was independent of the D-UVB dose measured in our geographical area. Due to the cross-sectional nature of the study, causality analysis is not permitted.

## 4. Discussion

As far as we know, this research is the first to explore the changes in 25(OH)D levels in pregnant women due to confinement related to the COVID-19 pandemic.

The results showed that there was a high frequency of VDD among pregnant women in our region (55.5%), which is consistent with previous reports [31,32]. This VDD prevalence was greatly influenced by the quarantine, with a significant increase in the SL group (77.8%), as a consequence of the decreased exposure to sunlight due to the in-house confinement in this cohort of participants. Half of the pregnant women in our study area have a vitamin D deficiency. Although these data are comparable to other studies, they are nevertheless important from a public health point of view. Our data describe a concerning reality that should be addressed.

25(OH)D is the preferred serum marker to assess vitamin D status, and it has a half-life of 15 days [33]. To adjust for seasonal irradiation, we included in our model an “environmental” variable, the D-UVB dose in biweekly intervals. It is accepted that vitamin D cutaneous synthesis is influenced by geographical latitude [6]. In fact, the term “vitamin D winter” was employed to describe the fact that exposure to winter sunlight in Boston (42.2° N) and Edmonton (52° N) will not promote vitamin D3 synthesis in human skin from November through to February (Boston) and from October through to March (Edmonton) [34]. A comprehensive analysis of seasonal and geographical variation in D-UVB in Europe has been recently published [7]. This D-UVB variation has been previously connected with 25(OH)D levels in Europe [30]. “Vitamin D winter” has been defined as the threshold of D-UVB dose below which (<1 kJ/m^2^) vitamin D synthesis is negligible [7,30]. In the southeast of Spain, the “vitamin D winter” ranged from December 2019 to mid-January 2020. According to the study by Khanna et al., despite the relatively moderate latitudinal range in Europe (35–64° N), large seasonal and regional variations in UVB radiation that can influence vitamin D production (D-UVB) were reported throughout the European continent. Winter vitamin D was <2 months in low European latitudes (and >7 months in high European latitudes). In our study, “vitamin D winter” ranged from December 2019 to mid-January 2020, which is consistent with the observations in low European latitudes (“vitamin D winter” lasting <2 months).

Our results revealed a decoupling of D-UVB dose and 25(OH)D levels during SL. While D-UVB doses ranged from 4.2 kJ/m^2^ to 8.5 kJ/m^2^ during in-house confinement period, 25(OH)D mean levels were lower than in real “vitamin D winter” (D-UVB <1 kJ/m^2^). The prevalence of VDD increased during strict confinement with respect to the “vitamin D winter”, even though ultraviolet B irradiation was at least four times higher. We could say that strict confinement produced a second “vitamin D winter” in the pregnant women in our study in an artificial way.

The skeletal muscle has been recently involved in vitamin D maintenance during “vitamin D winter”. Since no clear storage organ or tissue has been identified for vitamin D, it has been suggested that appropriate vitamin D status in winter can only be obtained by oral supplements. It has recently been shown that the main circulating metabolite of vitamin D, 25(OH)D, accumulates in skeletal muscle cells, which could constitute a functional reserve during the winter months [35]. We found no difference in BMI in relation to SL status. However, muscularity ranges widely, even at any specific level of BMI [36]. Skeletal muscle composition was not measured in the study participants; therefore, we cannot assess the role of this in the results that were obtained.

In addition to skin production, vitamin D-enriched diets and supplementation are also relevant sources of vitamin D. Access to food and medication was not significantly restricted during home confinement, and no special measures were taken to supplement the participants.

No other variables were significantly different in relation to SL, including GDM prevalence, BMI, and BMI group. Only triglycerides were significantly higher in the SL group, and nutritional and lifestyle items scored worst in the survey. In the adjusted logistic regression models, they did not significantly influence the POR for VDD. These results might be consistent with the confinement status. It is probable that some effects of lockdown, either behavioural or biological, may take longer to be apparent than others.

In the resulting adjusted binary regression model, SL was the most important factor explaining the abrupt increase in VDD prevalence. There were no other factors influencing these results (GDM, BMI group, or ethnicity). Therefore, in the case of a similar situation, an increase in the prevalence of VDD must be anticipated irrespective of these conditions.

In contrast to our finding, a study that included all outpatients older than 18 years old in a regional hospital in Verona (Italy) reported a similar prevalence of VDD during and after the lockdown period compared to the previous two years [37]. The pregnant women population of Elda (Spain) had a higher prevalence of VDD in the pre-lockdown period than those described in the study from Verona, but the result was similar to other maternal series [11,31,32]. The drop in 25(OH)D levels we found in SL has been described in Chinese children during home confinement due to the COVID-19 pandemic [38]. 

Some limitations should be recognised. Firstly, the results may not be generalisable due to the regionally based design of the study. Secondly, environmental data of the D-UVB dose in our area may not exactly reflect the individual solar exposure of participants. We employed TEMIS-ESA measured data as a surrogate of individual exposition [7,30]. Some studies stress the importance of individual UVB radiation measurement rather than assuming individual exposure [39]. This cross-sectional study did not take into account the amount or duration of sunlight exposure for each individual participant. The variations in sunlight exposure between the participants may have biased the results. In addition, finally, the current research was only based on a single determination of 25(OH)D levels, and did not consider any longitudinal changes in 25(OH)D levels. In a longitudinal study, the differences in vitamin D levels during pregnancy were reported, with a progressive increase in 25(OH)D levels during the pregnancy [40]. The cross-sectional design of the study prevents us from conducting a causality analysis. 

A strength in our study is serendipity. We had the “unique opportunity” to evaluate the effect of a rare event, such an in-house confinement, in a group of participants [18]. For ethical reasons, this condition could not be experimentally reproduced in any way.

## 5. Conclusions

Our results revealed that VDD prevalence in pregnant women was influenced by SL. This valuable information could guide us in the future to take action, such as with vitamin D supplementation, in the case that public officials order the population to stay indoors for any given reason.

## Figures and Tables

**Table 1:** Participants’ characteristics in relation to “Strict Lockdown” status (SL).

Characteristics	Non-Strict Lockdown (*n* = 715, 80.7%)	Strict Lockdown (*n* = 171, 19.3%)	*p*-Value
Sociodemographic characteristics			
Age (years), mean (SD)	32.0 (5.8)	31.7 (5.6)	0.48
Questionnaire (St. Carlos Study), mean (SD)			
Physical activity	−0.7 (1.4)	−0.8 (1.4)	0.26
Nutritional status	4.1 (3.6)	3.2 (3.9)	0.02 *
Lifestyle	3.3 (4.1)	2.3 (4.4)	<0.01 *
Maternal smoking habit, *n* (%)	79 (11.0)	12 (7.0)	0.12
Maternal hypothyroidism, *n* (%)	158 (22.1)	37 (21.6)	0.90
Ethnicity, *n* (%)			0.12
Caucasian	626 (87.7)	140 (82.8)	
South American	44 (6.2)	18 (10.7)	
Other	44 (6.2)	11 (6.5)	
Pregnancy-related characteristics			
SAP, first trimester (mmHg), mean (SD)	110.6 (11.7)	111.7 (11.6)	0.28
DAP, first trimester (mmHg), mean (SD)	68.8 (9.1)	68.7 (7.8)	0.78
VDD, *n* (%)	356 (50.1)	133 (77.8)	<0.01 *
GDM, *n (%)*	73 (10.2)	20 (11.7)	0.57
BMI, mean (SD)	24.7 (4.7)	25.2 (4.8)	0.25
BMI group, *n* (%)			0.12
Normal (<25)	441 (62.0)	91 (53.5)	
Overweight (25–30)	180 (25.3)	54 (31.8)	
Obesity (>30)	90 (12.7)	25 (14.7)	
Parity, *n* (%)			0.84
Primigravida	360 (50.3)	86 (50.3)	
2 pregnancies	270 (37.8)	62 (36.3)	
≥3 pregnancies	85 (11.9)	23 (13.5)	
History of caesarean section, *n* (%)	77 (10.8)	21 (12.4)	0.55
Gestational hypothyroidism, *n* (%)	158 (22.0)	37 (21.6)	0.90
Blood test, mean (SD)			
Vitamin D (ng/mL)	20.5 (8.9)	14.2 (6.7)	<0.01 *
C-reactive protein (mg/dL)	5.8 (6.1)	5.3 (4.9)	0.22
Ferritin (mg/dL)	24.6 (25.3)	24.5 (26.4)	0.96
Cholesterol (mg/dL)	223.4 (37.4)	228.4 (43.4)	0.18
HDL cholesterol (mg/dL)	76.0 (15.1)	75.0 (15.9)	0.44
LDL cholesterol (mg/dL)	113.8 (32.0)	115.5 (34.6)	0.58
Triglycerides (mg/dL)	173.4 (63.7)	193.5 (82.5)	<0.01 *
Fibrinogen (mg/dL)	399.5 (56.2)	391.1 (56.1)	0.09
Haemoglobin (g/dL)	11.6 (0.9)	11.6 (0.8)	0.64
Haematocrit (%)	34.1 (2.5)	34.4 (2.7)	0.23

* *p* < 0.05. Abbreviations: VDD, vitamin D deficiency; GDM, gestational diabetes mellitus; SAP, systolic arterial pressure; DAP, diastolic arterial pressure; BMI, body mass index.

**Table 2:** Association study. Prevalence odds ratio for VDD, estimated by crude and adjusted logistic regression models.

		Crude Model			Adjusted Model		
		POR	95%CI	*p*-Value	POR	95%CI	*p*-Value
Strict Lockdown:	No	1			1		
	Yes	3.5	(2.4–5.1)	<0.01 *	4.0	(2.7–5.7)	<0.01 *
D-UVB dose:					0.9	(0.9–0.9)	<0.01 *

* *p* < 0.05. Abbreviations: POR, prevalence odds ratio; CI, confidence interval; VDD, vitamin D deficiency; D-UVB, vitamin D ultraviolet B dose.

## Data Availability

Not applicable.
